# On the Alleged Action of Sulphate of Quinine on the Spleen; and upon a New Mode of Examining This Organ

**Published:** 1845-09

**Authors:** 


*On the Alleged Action of Sulphate of Quinine on the Spleen ; and upon a New Mode of Examining this Organ.*—Two years ago, in a memoir submitted to the Academy of Sciences, M. Piorry advanced the opinion, that sulphate of quinine, dissolved in a small quantity of sulphuric acid, and administered in moderate doses, acted so rapidly upon the spleen, that in the course of 40 *seconds* a notable diminution in the size of this organ took place. Since then, M. P. has repeated the statement, and at different times has exhibited the experiment in the wards of his hospital, in so short a time, as to convey to the mind an idea of illusion, (prêstige,) but not conviction. M. Gouraud having repeated the experiment, states, that in his hands it succeeded equally well, finding, that after the administration of a solution of sulphate of quinine, the region of the spleen acquires, in even less time than 40 seconds, a well marked clearness on percussion. " So much so," he says, " that the first idea that occurs to the mind is, that a considerable diminution in the size of the spleen has taken place." Doubts, however, arising in the mind of M. Gouraud, not as to the reality of the fact, but as to its explanation, he inquired, if the modification thus produced in the state of the parts, was really owing to a diminution in the size of the spleen, or not rather to dilatation of the stomach, produced by the sudden development or displacement of gaseous fluids. He tested the matter by experiment, and found, that on varying the fluid administered, using the same quantity of distilled water, with a few drops of sulphuric acid, without sulphate of quinine; then distilled water alone, lemonade, wine and water, or a common tisane, the same result, namely, disappearance of the spleenic dulness, was obtained, as after the exhibition of the sulphate of quinine.

" The correct conclusion," says M. G., " from these numerous facts is, that the ingestion into the stomach of a small quantity of fluid suffices to produce a development of gas so considerable, as to give a sonorousness almost tympanitic to the region of the spleen in 40 *seconds, or even less.*"

Another method of ascertaining the accuracy of this conclusion, is auscultation. If we apply the ear over the dull region of the spleen, at the instant a patient is swallowing a mouthful of water, we shall be struck with the loud sonorous gurgling produced by the fluid falling into the empty stomach, and can naturally explain the great sonorousness almost immediately afterwards produced in the gastro-spleenic region.

M. Gouraud does not confine himself to this first result, but generalising his observation, applies it not only to the hypertrophied spleen in fever, but also to the spleen in the natural state,—in this, following M. Piorry, who, in his memoir, states, "*it is no less extraordinary, that the spleen when healthy diminishes as rapidly under the sulphate of quinine, as it does when in a state of disease.*" This is so extraordinary a fact, that one must possess the conviction peculiar to M. Piorry, not to perceive, that what he wished to establish in his memoir, as to the properties of the sulphate of quinine, is, by it, completely and definitely overturned. Considering that in a certain class of subjects, in whom the spleen is healthy, the gastro-spleenic region, nevertheless, presents a dulness sufficiently great to lead at first to the idea, that there is a universal developement of the spleen, and having put forth the opinion that the dulness probably depends on the empty state of the stomach and transverse colon, whose walls lie in apposition, M. Gouraud asks, in what class of subjects is it, that this purely physiolocal phenomenon is perceived ? Inquire, and you will find, it is those who have not recently drank. In what class is the phenomenon absent? In those who, within the last quarter or half an hour, have swallowed some liquid." It is impossible not to admit the exactness of the facts and the reasoning employed, and from these follow results equally advantageous to science; first, the discovery of an error; and next, the acquisition of a fact, the importance of which, M. G. has not sufficiently insisted on, viz. the production and development of gas at the moment a fluid is taken into the stomach. We entirely coincide with M. Gouraud, in the following conclusion, viz. "That in order to decide with certainty that gastro-spleenic dulness is dependant on hypertrophy of the spleen, the degree of this dulness in the normal state, and under different physiological conditions, independent of disease, must be first ascertained. Before terminating his letter, the author points out one mode which has appeared of some value in appreciating the size of the spleen under certain circumstances, and which has not been alluded to in the article "Exploration of the Spleen," in the *Traite de Semeiologie;*—and that is to cause the patient to make a violent expiratory effort with the abdominal muscles. By the contraction of the diaphragm, all the organs below it are pushed downwards; and the spleen in particular, which descends more than an inch, and becomes quite distinct to the touch.

Having thus stated, as clearly and shortly as possible, the facts brought out in the letter of M. Gouraud, we ought also, perhaps, to give the answer of M. Piorry, and the reply by M. Gouraud; but the task is not an easy one; suffice it to say, M. Piorry has repeated the experiments with most fluids, and has not attained the results related by his opponent; he has also repeated those with a solution of quinine, and obtained the same effects as formerly. M. Gouraud has also repeated these, and again arrived at the conclusions above detailed; he concludes the discussion with these words: "I continue to maintain the general fact which I have announced, viz. that the disappearance of dulness in the spleenic region appears to be dependant on the ingestion of fluid, rather than on the action of sulphate of quinine." Between opinions so opposite, and facts so directly contradictory, it is imposible to decide; the determination lies in an appeal to new facts, observed and reported by those who are not personally interested in the question. Without leaning to one more than the other, we conclude with the words of M. Gouraud: "Practitioners, and those who have clinical opportunities, must decide the question; as for me, I will range myself on the side of truth, be it for or against me, whenever that truth is demonstrated; but it must be first demonstrated."—*Lond. and Ed. Month. Journ. of Med. Sci., from Journal des Connaissances Medico-Chirurgicales,* as quoted in *Gazette Medicale.*

